# Relationship Between the Use of Screens and the Motor Development of Children from 8 to 10 Years of Age

**DOI:** 10.3390/children12050584

**Published:** 2025-04-30

**Authors:** Jacqueline Páez-Herrera, Simón Álvarez-González, Sebastián López-Lara, Cristóbal Otero-Tricio, Waldo Rojas-Martínez, Sofía Salgado-Díaz, Rodrigo Yáñez-Sepúlveda, Juan Hurtado-Almonacid

**Affiliations:** 1 eFidac Research Group, Physical Education School, Pontificia Universidad Católica de Valparaíso, Valparaíso 2340025, Chile; simon.alvarez.g@mail.pucv.cl (S.Á.-G.); sebastian.lopez.l01@mail.pucv.cl (S.L.-L.); cristobal.otero.t@mail.pucv.cl (C.O.-T.); waldo.rojas.m@mail.pucv.cl (W.R.-M.); sofia.salgado.d@mail.pucv.cl (S.S.-D.); juan.hurtado@pucv.cl (J.H.-A.); 2 Faculty of Education and Social Sciences, Universidad Andres Bello, Viña del Mar 2520000, Chile; rodrigo.yanez.s@unab.cl

**Keywords:** motor skills, electronic devices, sedentary behavior

## Abstract

Background: The considerable increase in screen exposure causes negative effects on the motor development of boys and girls, which results in a decrease in the level of physical activity and an increase in sedentary behaviors. In Chile, this problem has been widely addressed in adolescence; however, it is necessary to address it in childhood and early adolescence with a preventive approach. Objectives: To know the relationship between the use of screens and motor development in children. Methods: The sample was composed of 206 boys and girls (girls = 126 and boys = 80). The ages of the students ranged from 8 to 10 years old with a mean in months of 89.6 ± 7.54. The Gross Motor Development-2 was applied to identify motor development, screen time was measured using the Questionnaire to measure physical activity and sedentary behavior. Results: Girls have a greater weekly time than females in the use of consoles (*p* = 0.004). Girls and boys who are in the very poor, poor, and low average levels of motor development have more time in front of TV, PC, consoles, and total time with screens. There is an inverse relationship between the level of motor development and weekly screen time (r = −0.252). Conclusions: Screen exposure has a negative effect on the motor development of boys and girls. Although there is compliance with World Health Organization recommendations for screen exposure, the motor development of the participants is mostly very poor, poor, and low average.

## 1. Introduction

During the last few years, there has been a considerable increase in the use of screens, becoming a daily and habitual activity of people [1]. The increasing exposure to screens has had negative effects on the motor development (MD) of boys and girls, constituting a barrier to the development of motor creativity, MD, and physical activity level [2]. This is due to the fact that, increasingly at younger ages, boys and girls are exposed to electronic devices, generating a concern in the area of health [3] because as the time they spend in front of different screens increases, their sedentary behaviors also increase, which are closely associated with being overweight and obese [4,5]. As a result of this, the World Health Organization (WHO) has designed recommendations on screen time, where it establishes that infants and children from 1 year of age should not engage in sedentary activities in front of screens [6]. On the other hand, it is recommended that infants aged 2 to 4 years should not exceed a maximum of 1 h in front of these devices, always emphasizing that the less time they spend in front of screens the better [6]. For its part, the evidence suggests that in much of the world, the WHO recommendations are ignored [7,8]. The literature shows that children and adolescents between 8 and 18 years of age spend more time in front of screens than in other activities, spending about 1/3 of their day in front of these devices [9]. Adolescents are the groups who most commonly present this behavior, failing to comply with the screen time recommendations proposed by the WHO [10]. Excessive screen time brings with it harmful effects on the health of school-age boys and girls; emotional, sleep, and behavioral problems affect growth, cognitive, and motor development of school-age boys and girls [1,11].

The MD is a continuous and progressive process of change in human motor skills and abilities [12,13], resulting from an interaction between genetic, environmental, and learning factors [14,15,16]. During childhood, MD is related both to brain development and the acquisition of motor skills [17,18], as well as to the development of social and emotional skills [19,20]. Thus, as boys and girls acquire new motor skills, they also improve their ability to resolve problems, express emotions, and form social relationships [15,21]. Likewise, having an adequate level of motor development and competence brings with it higher levels of physical activity and thus greater health benefits during childhood and adolescence [22]. As fundamental movement skills (e.g., running, jumping, throwing, among others) develop favorably, not only is there an improvement in motor performance, but there is also a reinforcement and maintenance of an active lifestyle [23].

In addition, Hulteen et al. [24] points out that when cultural and environmental practices are enriching, focusing on play [16,25], with the alternation of structured and unstructured physical activity [26], the level of MD is favored [27]. Meanwhile, there is a positive relationship between MD and child and youth health, since participation in physical activity, perceived physical competence, physical condition, and a healthy weight status are favored, which has an impact on the decrease in sedentary behavior, a low cardio-metabolic risk and a better physical condition, improved flexibility, self-esteem, cognitive development, school readiness, and academic achievement [28]. Meanwhile, when children increase their sedentary activities such as watching television, playing video games, or spending time on the phone while lying down or sitting, they reduce their opportunities to be physically active and to meet the physical activity (PA) levels established by the WHO [5,6,29]. Currently, about 80% of adolescents do not meet these minimum PA recommendations [5]. Low levels of movement and motor exploration caused by physical inactivity can severely compromise the MD of children [30], and this direct relationship can persist throughout a person’s life [22,31]. When the relationship is positive, boys and girls with a higher level of motor development have a higher level of physical activity [28,32,33,34]. Therefore, although there is evidence on the association between exposure to screens and the level of MD in young children, it is still necessary to continue gathering evidence on the influence of the devices on the trajectory of older children [35]. The use of screens diminishes and alters sensorimotor experiences, as visual and auditory sensory inputs are over-stimulated [36], causing a considerable decrease in the motor activity of locomotion and object control, which is developed preferentially in three-dimensional space [37]. The literature indicates that there is growing concern among specialists and parents about the impact of screen use on boys’ and girls’ development [38,39,40].

Evidence indicates that the effect of screens has been extensively studied in late adolescence; however, it needs to be addressed in childhood in a preventive manner [39,41,42].

Based on the above, the purpose of this study is to determine the relationship between the use of screens and motor development in boys and girls between 8 and 10 years of age.

## 2. Materials and Methods

As for the method, this study is situated in the quantitative paradigm, has a correlational scope, is non-experimental and is cross-sectional [21].

### 2.1. Participants

The sample was obtained non-probabilistically and by convenience; a total of 206 students participated (girls = 126 (61.17%) and boys = 80 (38.83%)), who were in the 3rd and 4th years of elementary school. The ages of the students ranged from 8 to 10 years old with a mean in months of 89.6 ± 7.54 (SD).

The inclusion criteria for selecting participants included having a minimum class attendance of 70% and not having a pathological condition that prevented the performance of PA. Participants with lower attendance were excluded. Students who participated in this study attended mandatory physical education classes.

### 2.2. Procedure

The application of the protocols was carried out considering the ethical principles for research on human beings proposed by the Declaration of Helsinki [43] and the procedural and documentation suggestions of the Research Department of the Pontificia Universidad Católica de Valparaíso (Chile) through the Scientific and Bioethical Ethics Committee (BIOEPUCV-H-456-2021). Authorization was requested from the authorities of the educational establishments to carry out the research project. Once the authorities approved the research project, the informed consent form was sent to the parents and/or responsible guardians, informing them of the objectives and scope of the study, in order to authorize the participation of their boys and girls in the study. Subsequently, during the Physical Education classes, the motor development test (TGMD-2) was applied, starting with the locomotion tests and continuing with the object control tests. The motor development test was administered by a total of 12 examiners. The team was composed of the two teachers responsible for the project, and ten students of the physical education pedagogy career assigned to the eFidac research laboratory. This last group underwent a three-week training course, consisting of a theoretical instance (week 1) where they were able to learn the theoretical basis of the test, as well as the scoring and classification scales. Subsequently, they underwent two practical training sessions (weeks 2 and 3), where they were able to learn the protocols of the locomotion and object control tests, applying them to a small group of boys and girls of the ages of the participants in this study. For the application of the test, the total number of evaluators was divided into two groups (6 members each), each of them was responsible for the locomotion and object control tests, and each group was led by the project leaders.

This study also included the application of a parental report questionnaire on behaviors associated with physical activity and sedentary lifestyles. This instrument was answered by one of the two parents at the educational establishment. For this purpose, a meeting was arranged with the parents who authorized the participation of their sons and daughters. In order to resolve concerns when answering the instrument, a group of three researchers participated in this meeting. The researchers responsible for the application of the instrument were trained two weeks prior to its application. First, they thoroughly reviewed the instrument and resolved any concerns about terms and concepts. Subsequently, they simulated its application in a sample of adults with similar characteristics to the guardians and/or parents participating in this study.

### 2.3. Instruments

To identify motor behaviors, the TGMD-2 [44] was used, which presents validity indexes of 0.93 for language clarity and 0.91 for relevance [21]. The purpose of this instrument is to identify motor development in children between 3 years and 10 years 11 months of age, categorizing motor behaviors into seven categories: very poor, poor, low average, average, above average, superior, and very superior. The test identifies these categories according to gender and years of age in months, considering the evolutionary motor development of children. Gross motor skills are evaluated and grouped into two subsets: locomotion skills (run, gallop, hop, leap, horizontal jump, and slide) and manipulation skills (striking a stationary ball, stationary dribble, catch, kick, overhead throw, and underhand roll). This is in order to generate three grades: one for locomotor development, another for the manipulation area, and the third for gross motor development in general. For the application of the tests, a space of 30 m of flat surface was chosen, following the guidelines established in [21,43,44]. The locomotion tests as well as the object control tests were arranged in successive stations, where all the participating children performed them individually. The locomotion tests were organized as follows: run, gallop, hop, leap, horizontal jump, and slide. The object control tests were arranged in the following order: striking a stationary ball, stationary dribble, catch, kick, overhead throw, and underhand roll.

Each gross motor skill includes three to four behavioral components that are presented as performance criteria, where a score of 1 is recorded if it is performed correctly and 0 otherwise. After applying the test and adding the two attempts per test of the two subtests, the scores obtained should be analyzed with the conversion table according to the age in months of the child, which provides a score called Standard Scores that describes a Gross Motor Quotient where it ends with the description of the range in the categories of very superior 130, superior 121–130, above average 111–112, average 90–110, below average 80–89, poor 70–79, and very poor 70 [45,46].

To determine screen time, the Questionnaire for measuring physical activity and sedentary behavior in pre-school children (CMAFYCS) was used, which is a questionnaire for measuring physical activity and sedentary behaviors in boys and girls from preschool to fourth grade by Camargo et al. [47], with an internal consistency under Cronbach’s alpha of 0.64 considered good [48]. The questionnaire records 12 items of activities performed, considering the previous week. It is a report made by the parents and/or guardians of the children. It records the time spent for each activity every day of the week. It has three dimensions, the first of characterization, the second of physical activity, and the third of sedentary behaviors that are classified as activities that require minimal or no movement and require little effort. In relation to screen time, the questionnaire has 5 dimensions: time spent reading, watching TV in the bedroom, time spent in front of the TV, time spent on the computer (playing or listening to music), and time spent on consoles or video games. The answer for each item is dichotomous: yes or no, and under an affirmative answer the time dedicated to the activity is reported in minutes and hours during each day of the week.

To establish compliance with screen time, the WHO and American Academy of Pediatrics guidelines were followed, which suggest excessive exposure when it exceeds two hours [48].

### 2.4. Data Analysis Technique

For data analysis, the parametric Mann-Whitney U test was used to compare ordinal variables (MD) with nominal variables (sex), the chi-squared test was used for nominal variables (WHO Compliant) with nominal variables (sex), and the Student’s *t*-test for independent samples was used to analyze differences in measures of central tendency. All were tested for statistical significance at a 95% confidence level (*p* < 0.05).

For correlational analysis, the Spearman Rho test was applied (ordinal variables–numerical variable) to determine the existence of an association between MD variables and screen time, and to test for statistical significance at a 95% confidence level (*p* < 0.05).

IBM SPSS Statistics 25 software (New York, NY, USA) was used to perform these analyses.

## 3. Results

Table 1 presents, rather than analyses the distribution of the participants for the locomotion, object control, and total MD tests. In this respect, it can be seen that 52% of the participants (girls and boys) are located in the very poor, poor, and low average levels. In relation to the object control tests, 42.2% of the participating boys and girls are in these categories. A similar situation is observed in the total MD (61.1%). Regarding the superior or very superior categories, no student is located in these levels, regardless of sex. Comparing the results between girls and boys, a significant difference is found in the dimensions of object control and total motor development (*p* < 0.001) where girls present a better level of development.

Table 2 shows the means and standard deviations according to the sex of the participants and the time spent daily using screens of different devices. The highest daily usage time is spent watching television and playing games with consoles. When comparing according to sex, the time spent daily using the console device is greater in boys (15.91 min per day), and statistically significant differences can be observed (*p* value = 0.004).

Table 3 shows the frequencies of screen use according to WHO recommendations. Thus, the female participants in this study present a high percentage of compliance with the WHO recommendations for all devices (TV = 96.03%; PC = 79.36%; Consoles = 89.68%; Screens = 84.12%), presenting statistically significant differences compared to their female peers who do not comply with the WHO recommendations. When observing these behaviors in male participants, the situation is similar to that of the girls (TV = 96.25%; PC = 83.75%; Consoles = 71.25%; Screens = 78.75%). However, there is a higher percentage of female participants who meet the recommendations for PC and console usage time (Consoles = 18.43%; Screens = 5.37%). When comparing groups according to sex and the total group, who meet the WHO recommendations for daily use time of different devices, a statistically significant difference is observed in favor of those participants who comply with the recommendations.

Table 4 shows the correlation between the motor development of the subjects and compliance with the recommendations for daily use of screen time. It is possible to observe that regardless of compliance with WHO recommendations for the use of devices, a high percentage are at very poor, poor, and low average levels of motor development (61.14%). However, when associating the level of total motor development of the children with compliance with the WHO recommendations for the use of devices, a weak inverse negative correlation (r = −0.236) and statistically significant (*p* = 0.001) was observed. This indicates that as more participating children increase their level of compliance with the recommendations, there are also fewer who have above average or very high levels of motor development.

Table 5 shows the correlation between the motor development of the subjects and the time spent daily using screens, where the relationship is inverse (r = −0.252), i.e., the more time spent using screens, the lower the motor development. This situation is exacerbated especially in those participating children located in the very poor, poor, and low average levels of motor development, who use more TV time per week (125.03 min per week); PC (34.29 min per week), and consoles (13.44 min per week). The same situation is evident when observing the total time of weekly use of any device, where the participants located in the very poor, poor, and low average levels present greater time of weekly use of electronic devices.

## 4. Discussion

The aim of the study was to determine the relationship between screen use and motor development in boys and girls aged 8 to 10 years. Thus, screen time (TV, consoles, PC, and total time), the level of total motor development, object control, and locomotion were examined in boys and girls between 8 and 10 years old. First of all, the results indicated that girls had higher scores in the object control, locomotion, and total MD tests. This situation is similar in other studies with Chilean population where the results indicated that girls, between 8 and 10 years old, presented a higher level of MD for locomotion, object control, and total MD tests, compared to their male peers [43,44,45].

Likewise, Páez et al. [46] when relating the level of physical activity of the parents with the MD and Body Mass Index of their children, they found that girls were located to a lesser extent in the lower categories of “very poor and poor MD”, in relation to their male peers. Likewise, girls present a lower daily and weekly time of exposure to devices (PC, consoles, and other screens) compared to their male peers.

These results could be explained by the fact that the group of girls in this study had less daily and weekly time of exposure to devices (PC, consoles, and other screens) compared to their male peers. Thus, high screen time is associated with poor MD [49] especially with manipulative skills [39,50]. This situation is explained by the fact that the more time spent in front of screens, the more physical activity is affected, and consequently a considerable decrease in motor skills is seen [51].

Likewise, the results of the study reported that there is an inverse compensation between MD and screen time, that is, the less time children spend in front of the screen, the better their MD. The same situation occurred in a population of Canadian children, where those who obtained lower scores in motor development tests, particularly those associated with locomotion, presented more time in front of screens [52,53], precisely Martins et al. [54] indicate that screen time is an environmental factor that negatively influences the performance of basic motor skills. Meanwhile evidence suggests that those children who spend more time exposed to screens at the age of 4 years also do so at the age of 5 and 7 years, also presenting a negative relationship in motor competence tests [55]. This situation is exacerbated at the age of 8 years, where children begin to spend more time in front of screens, dedicating practically a third of their day to using electronic devices [9].

Those children who spend more time daily and weekly exposed to screens are less likely to reach adequate zones in the development of their motor skills, and consequently for their physical condition [51]. Likewise, this exposure to screens has a direct impact on the possibilities of interacting with and exploring their environment [55], with direct repercussions on the development of their motor skills [56].

The considerable increase in screen exposure was accentuated from 2020 onwards, which is due to the confinement and consequently the development of online classes, as children were forced to spend more time at home, making the use of these devices more common [1].

When observing the behavior of the participants in terms of compliance with the WHO recommendations regarding daily and weekly screen time, the results indicated that a higher percentage of children comply with the recommendations established by the WHO for screen exposure time. Similar results were evidenced in a study with Canadian children who reported a compliance of 50.5% [51]; however, they did not meet the recommendations for general physical activity and hours of sleep. This is explained by the fact that this triad of behaviors associated with quality of life are determined by the environment and depend to a large extent on the integrated approach with which these habits are developed [56].

This leads to an increase in sedentary time, less physical activity, and a decrease in the level of MD [21,28,29]. Those children who are exposed early to more time in front of screens have a greater risk of stabilizing this behavior over time, causing a decrease in MD [52]. However, there is evidence to suggest that boys and girls may substitute different types of sedentary behavior, leaving the association between the use of electronic devices and motor development unclear [57].

Among the limitations, we highlight the lack of an objective assessment of physical activity levels, which would have allowed us to establish a broader profile of the impact of screen time and, therefore, its influence on motor development. Furthermore, we believe it would have been interesting to consider active and passive screen time, as well as to incorporate weekend screen time.

The time parents of participating children spend in front of screens should also have been considered in order to analyze the phenomenon from a more ecological perspective.

Regarding the study’s strengths, we can highlight that this research primarily considers a group that has not been fully explored (children aged 8 to 10) when establishing the relationship between screen use and motor development, since information has generally been collected from preschool children (aged 3 to 5). Likewise, the focus has traditionally been on the relationship between screen time and physical activity levels, so research of this nature offers new possibilities for study. Furthermore, it would be interesting to advance the establishment of national standards (in the context of Chile) on screen time, since the cultural reality, organization, and dynamics of families differ from other contexts. Similarly, establishing national standards on screen time for children would allow for greater coverage of variables to be considered when designing public policies that may help counteract the sedentary lifestyle and physical inactivity among children in Chile.

## 5. Conclusions

The study reveals that screen use in boys and girls is associated with motor development that is mostly at “very poor” and “fair” levels. While a high percentage of boys and girls meet WHO recommendations on screen time, the majority still have deficiencies in their motor skills. The inverse correlation observed between screen time and motor development suggests that while more screen time could be related to lower motor performance, more research is needed on the association between the two variables.

It is necessary to promote a balance between screen use and physical activity by promoting games and exercises that stimulate DM. Furthermore, further research is recommended to delve deeper into the relationship between screen use and DM, as well as to identify effective strategies to help improve DM in children. This research may provide useful information for parents, educators, and school staff to jointly address the increasing amount of screen time and the variety of devices, as well as the potential negative consequences for children’s physical, motor, and cognitive development.

## Figures and Tables

**Table 1:** Frequency distribution of locomotion, manipulation, and general motor development levels according to sex.

	Girls (n = 126)	Boys (n = 80)	Total (n = 206)	*U*	*Z*	*p*-Value ^1^
**Locomotion**						
Very Poor n (%)	7 (5.6)	9 (11.3)	16 (7.8)	4545.0	−1.26	0.205
Poor n (%)	11 (8.7)	12 (15.0)	23 (11.2)			
Low Average n (%)	47 (37.3)	23 (28.8)	70 (34)			
Average n (%)	56 (44.4)	34 (42.5)	90 (43.7)			
Above Average n (%)	5 (4.0)	2 (2.5)	7 (3.4)			
Very Superior—Superior n (%)	0 (0)	0 (0)	0 (0)			
**Object Control**						
Very Poor n (%)	4 (3.2)	5 (6.3)	9 (4.4)	3399.0	−4.31	<0.001.
Poor n (%)	14 (11.1)	19 (23.8)	33 (16)			
Low Average n (%)	21 (16.7)	24 (30)	45 (21.8)			
Average n (%)	78 (61.9)	32 (40)	110 (53.4)			
Above Average n (%)	9 (7.1)	0 (0)	9 (4.4)			
Very Superior—Superior n (%)	0 (0)	0 (0)	0 (0)			
**Motor Development Total**						
Very Poor n (%)	8 (6.3)	12 (15)	20 (9.7)	3506.0	−3.85	<0.001.
Poor n (%)	14 (11.1)	24 (30)	38 (18.4)			
Low Average n (%)	46 (36.5)	22 (27.5)	68 (33)			
Average n (%)	53 (42.1)	21 (26.3)	74 (35.9)			
Above Average n (%)	5 (4.0)	1 (1.3)	6 (2.9)			
Very Superior—Superior n (%)	0 (0)	0 (0)	0 (0)			

^1^: *p* < 0.05. U Mann–Whitney.

**Table 2:** Time (minutes) of daily weekly use of device screens (TV, computer, consoles and total screens).

	Girls (n = 126) $\bar{X}$ ± DS	Boys (n = 80) $\bar{X}$ ± DS	Total (n = 206) $\bar{X}$ ± DS	*p*-Value ^1^
Television Time	46.84 ± 48.24	46.52 ± 33.88	46.72 ± 43.14	0.958
Computer Time	9.44 ± 17.88	10.86 ± 21.9	9.99 ± 19.51	0.612
Console Time	7.55 ± 17.42	15.91 ± 23.81	10.78 ± 20.50	0.004 *
Total Screen Time	63.81 ± 54.79	72.31 ± 47.52	67.50 ± 52.18	0.204

^1^: * *p* < 0.05. *t* Student.

**Table 3:** Compliance with the WHO indications of 120 min daily use of different screens according to sex.

Screen Type	Woman (n = 126)		X^2^ (DF)	*p* ^1^	Man (n = 80)		X^2^ (DF)	*p* ^1^	Total (n = 206)		X^2^ (DF)	*p*-Value ^1^
	Yes	No			Yes	No			Yes	No		
TV	121 (96.03)	5 (3.96)	106.7 (1)	<0.001	77 (96.25)	3 (3.75)	68.4 (1)	<0.001	198 (96.11)	8 (3.88)	175.2 (1)	0.001
PC	100 (79.36)	26 (20.63)	43.4 (1)	<0.001	67 (83.75)	13 (16.25)	36.4 (1)	<0.001	167 (81.06)	39 (18.93)	79.5 (1)	0.001
Consoles	113 (89.68)	13 (10.31)	79.3 (1)	<0.001	57 (71.25)	23 (28.75)	14.1 (1)	<0.001	170 (82.52)	36 (17.47)	87.1 (1)	0.001
Displays	106 (84.12)	20 (15.87)	58.6 (1)	<0.001	63 (78.75)	17 (21.25)	35.4 (1)	<0.001	169 (82.03)	37 (17.96)	84.5 (1)	0.001

^1^: *p* < 0.05. Chi Square. X^2^: degrees of freedom.

**Table 4:** Correlation between motor development and compliance with screen use recommendations.

	Very Poor (n)	Poor (n)	Low Average (n)	Average (n)	Above Average (n)	Superior and Very Superior (n)	*p*-Value ^1^
Yes WHO Compliant	15 (7.28)	26 (12.62)	53 (25.72)	70 (33.98)	5 (2.42)	0 (0.0)	0.001 (r = −0.236)
Not WHO Compliant	5 (2.42)	12 (5.82)	15 (7.28)	4 (1.94)	1 (0.48)	0 (0.0)	

^1^: *p* Spearman’s rho; r: correlation coefficient.

**Table 5:** Correlation between motor development and weekly screen time.

Motor Development	Time TV $\bar{X}$ ± SD	Time Pc $\bar{X}$ ± SD	Time Console $\bar{X}$ ± SD	Total Screen Time $\bar{X}$ ± SD	*p*-Value ^1^
Very Poor (n = 20)	43.96 ± 35.25	19.07 ± 28.88	11.53 ± 13.90	74.57 ± 40.85	<0.001 (r = −0.252)
Poor (n = 38)	64.83 ± 76.35	11.93 ± 19.16	13.75 ± 22.56	90.52 ± 76.28	
Low Average (n = 68)	48.72 ± 32.05	9.87 ± 20.39	15.04 ± 26.84	73.64 ± 51.85	
Average (n = 74)	36.72 ± 26.63	6.89 ± 15.11	5.08 ± 10.50	48.70 ± 31.95	
Above Average (n = 6)	41.78 ± 15.55	7.14 ± 17.49	11.42 ± 25.95	60.35 ± 35.45	
Superior and very Superior (n)	0	0	0	0	

^1^: *p* Spearman’s rho; r: correlation coefficient.

## Data Availability

The original contributions presented in the study are included in the article, further inquiries can be directed to the corresponding author.
